# Sex Differences in HIV Infection

**DOI:** 10.1007/s11904-018-0383-2

**Published:** 2018-03-05

**Authors:** Eileen P. Scully

**Affiliations:** Division of Infectious Diseases, Department of Medicine, Johns Hopkins University School of Medicine, Pre-Clinical Teaching Building, Suite 211, 725 N Wolfe Street, Baltimore, MD 21205 USA

**Keywords:** HIV, Sex, Inflammation, Prevention, Pathogenesis, Cure

## Abstract

**Purpose of Review:**

This review will outline the multilevel effects of biological sex on HIV acquisition, pathogenesis, treatment response, and prospects for cure. Potential mechanisms will be discussed along with future research directions.

**Recent Findings:**

HIV acquisition risk is modified by sex hormones and the vaginal microbiome, with the latter acting through both inflammation and local metabolism of pre-exposure prophylaxis drugs. Female sex associates with enhanced risk for non-AIDS morbidities including cardiovascular and cerebrovascular disease, suggesting different inflammatory profiles in men and women. Data from research on HIV cure points to sex differences in viral reservoir dynamics and a direct role for sex hormones in latency maintenance.

**Summary:**

Biological sex remains an important variable in determining the risk of HIV infection and subsequent viral pathogenesis, and emerging data suggest sex differences relevant to curative interventions. Recruitment of women in HIV clinical research is a pathway to both optimize care for women and to identify novel therapeutics for use in both men and women.

## Introduction

A combination of environmental factors, host genetics, and viral features determines the acquisition and pathogenesis of HIV infection. Some of these features, such as host HLA genotype, have been delineated, but the diversity of clinical manifestations of HIV suggests multiple sources of variation that are, as yet, undefined. Biological sex, with a distinct genetic complement, hormonal environment, and behavioral and social context, is a substantial contributor to heterogeneity in host responses. Research defining sex differences serves a dual purpose: first, defining sex-specific responses will insure that interventions have efficacy in both men and women, and second, differences may highlight pathways that can be modulated in both sexes to optimize treatment and prevention and curative interventions.

Clinical studies to isolate the effects of biological sex are challenging, but work to date has yielded important insights. This review will address sex-specific features of HIV prevention, pathogenesis, and cure research, and then outline potential biological mechanisms for these differences. Finally, barriers to research on sex differences and to enrolling women in clinical trials are discussed, along with the opportunities to circumvent these obstacles.

## Prevention

### Sex-Specific Acquisition Risks

The risk of HIV seroconversion per heterosexual act is estimated to be approximately twofold higher for the female compared to male partner [1], with multiple contributing factors. The unique characteristics of the female genital tract as compared with rectal and penile mucosal surfaces confer differences in transmission risk. Inflammation at the cervicovaginal mucosa lowers the barrier to HIV infection [2–5], and both the vaginal microbiome itself [6] and sexually transmitted infections [7–11] are important determinants of the levels of local inflammation. The association of depot medroxyprogesterone (DMPA) hormonal contraception with enhanced risk of infection (hazard ratio of 1.4) [12–14] underlines the sex-specific risk associated with hormone exposure, which also impacts the vaginal microbiome. Clearly, these factors have distinct manifestations in the male and female genital tracts and these basic differences have important implications for prevention interventions discussed below.

### Vaccine Responses

Sex differences in both adverse effects and the efficacy of protective responses to vaccination are well described [15]. These differences are of clinical significance as seen in the higher rates of vaccine-associated severe viscerotropic yellow fever disease in women [16, 17] and the HSV glycoprotein vaccine that was protective only in women [18]. The mechanisms driving these differences are not totally clear; no specific immunologic correlate was reported for the sex differences in the HSV vaccine trial [18] although subsequent work suggested that specific epitopes may be preferentially recognized in women [19]. Systems biology analysis of gene expression profiles after yellow fever vaccine identified sex-specific programs of gene induction [20], highlighting the potential for studies of sex differences to identify correlates of successful protection. In HIV vaccine trials, there has not been clear evidence of sex differential effects. In the RV144 study, protective efficacy was estimated 25.8% in men (*n* = 4875) and 38.6% in women (*n* = 3085), with no statistical difference associated with sex [21]. In terms of immune correlates of protection, differences in humoral and cell-mediated immune responses have been seen in multiple vaccines [20]. Mechanistically, there is evidence for more potent induction of inflammatory pathways in cytotoxic T cells from women [22]; sex comparison of the magnitude and breadth of T cell responses induced by vaccines would be of interest. Likewise, there is data to suggest that somatic hypermutation is enhanced by estrogen [23] and that antibody glycosylation patterns are influenced by sex [24] suggesting that biological sex may influence both antibody affinity and non-neutralizing functions.

Moving forward, sex-specific analyses of both efficacy and immune correlates of protection should be leveraged to enhance responses. For example, sex-specific induction of type 1 interferons or the inflammasome might indicate a role for specific adjuvanting strategies in men versus women. Given the challenges of vaccine development, all avenues for optimization bear consideration.

### Pre-Exposure Prophylaxis

In the absence of an effective vaccine, pharmacologic strategies have become a critical adjunct to the prevention of transmission. Notably, despite initial studies showing high levels of efficacy for PrEP in men who have sex with men [25] and in serodiscordant couples [26], studies of PrEP exclusively in women showed no efficacy, results that were attributed to very low adherence to study drug [27, 28]. Clinical pharmacology studies have highlighted differences in drug concentration at the rectal mucosal and cervicovaginal tissues [29] that may obligate different levels of adherence in women versus men to maximize effectiveness. To circumvent this, topical delivery designed for the vaginal microenvironment is another potential route to modulate risk of infection in women; the CAPRISA 004 study reported a 39% risk reduction with tenofovir gel [30], although the VOICE study, which was limited by low adherence, did not show efficacy in the vaginal gel arm [27]. The topical approach using a vaginal ring preparation of the novel antiretroviral dapivirine has recently demonstrated a significant but modest reduction in the risk of HIV acquisition (27–31%) [31, 32]. Importantly, recent work has shown that adherence is not the only challenge to the topical approach. Local metabolism of tenofovir itself by components of the vaginal microbiome is associated with reduced efficacy of protection [33]. As studies defining the effects of topical exposure at the rectal mucosa have suggested that tenofovir may increase certain inflammatory mediators [34], specific assessment of the in vivo cervicovaginal effects is also warranted. Further studies are necessary to define the optimal approach to risk reduction in both men and women; advantages of topical preparations must be considered in light of adherence challenges, and careful studies are necessary to fully define sex-specific modulators of efficacy at the sites of acquisition. Taken together, the data suggest that there are sex-specific features of risk perception and medication adherence, along with critical differences in pharmacologic properties and the microenvironment at sites of acquisition in men and women. Considering these differences will be critical in the design and implementation of chemoprophylaxis strategies.

## Pathogenesis

### Disease Progression

Sex is a clear contributor to disease pathogenesis in multiple infectious diseases [35], and HIV follows this paradigm. Across most studies, women have lower HIV viral loads early during infection but despite this difference, disease progression is comparable between the sexes [36–46]. Substantial differences in immune activation may underlie this apparent paradox; women have higher CD8^+^ T cell activation at a given level of HIV viremia, corresponding to activation seen in men at one log_10_ higher viral load [47]. Similarly, the expression of interferon-stimulated genes was higher in women when controlling for HIV viral load [48]. Given the role of immune activation in driving HIV disease progression [49, 50] and in comorbidities that emerge during effective ART [51, 52], the sex difference in immune setpoint likely has clinical consequences.

In selected individuals, HIV disease progression is attenuated, with either spontaneous control of viral replication in the absence of drug therapy [53–55] or sustained viral suppression after interruption of ART (post-treatment controllers; PTCs) [56]. The factors that allow natural control of HIV are not fully defined but include host genetics, highly efficient immune responses, and in select cases, viral fitness [53–55]. Cohort studies have reported that women are more likely to be categorized as spontaneous controllers of HIV [57, 58] although the determinants of this advantage have not been elucidated. Similarly, women are overrepresented in cohorts of post-treatment control: women were 36% of PTCs, 43% of low viremia patients (viral load between 50 and 500), and only 14% of post-treatment non-controllers in one study [59]. Again, sex-specific mechanisms of protection have not been defined within this group, and it should be noted that the total numbers evaluated are very low. Thus, although limited by biases in case finding, women more frequently demonstrate phenotypes of viral control. This suggests that identifying sex determinants of immune response and viral setpoint may shed light onto features of a successful host response.

### Response to Treatment

Consistent with sex differences in pharmacokinetics/pharmacodynamics, drug metabolism, body composition, and drug distribution, the rates of adverse reactions with the early generation of antiretroviral therapies showed sex variation [60, 61]. Efforts to analyze these differences are hampered by the limited enrollment of women in trials of new therapeutics [62]. In response to this challenge, the GRACE (Gender, Race And Clinical Experience) trial specifically enrolled women to determine the sex-specific efficacy of a darunavir-based ART regimen [63] and yielded critical insights into the barriers to participation by women (discussed further below) [64]. Recent subgroup analyses of therapeutic trials have largely demonstrated similar efficacy in men and women, consistent with the improved therapeutic index of modern antiretrovirals [65–67]. However, unanticipated effects of antiretrovirals, such as the recently reported weight gain associated with integrase inhibitor regimens in a predominantly male cohort (14% women in integrase inhibitor subgroup) [68], should be carefully evaluated for sex effects. In addition, the response to treatment as measured by CD4^+^ T cell recovery has been reported to favor women, although with unclear implications for immune competence [69]. Complications of immune reconstitution such as the immune reconstitution inflammatory syndrome (IRIS) have not been reported to have a particular sex predilection. However, this is difficult to clearly establish given the heterogeneity in case definitions of IRIS, bias for women to be enrolled in resource-limited settings, and lack of disaggregation of data by sex in some larger studies.

Treatment-induced changes in biomarkers of inflammation also show discordance; in one cohort, women had higher baseline high-sensitivity C reactive protein (hsCRP) levels and less change with therapy, along with higher levels of soluble CD163, a marker of monocyte activation [70]. Other cohorts have reported similar differences in response to treatment, although inconsistent differences in baseline levels [71]. Further work will be necessary to dissect the direct contribution of HIV and ART as compared with concurrent inflammatory stimulators such as coinfections and smoking, and modulators such as sex hormones given the potential for direct effects of estrogen on some markers such as CRP [72]. Overall, women and men can both achieve viral suppression with ART but differences in residual immune activation and reconstitution may remain, with consequences for comorbid conditions.

### Non-AIDS Morbidity and Mortality

With the advent of effective ART, morbidity and mortality among people living with HIV has shifted to non-AIDS events including cardiovascular disease, cancer, and neurocognitive dysfunction, many of which are driven by inflammatory consequences of HIV infection. Biological sex is one contributor to the multifactorial determinants of these comorbidities [52]. The excess risk of cardiovascular events in people living with HIV [73] is amplified in women [74] and linked to higher levels of circulating markers of monocyte activation [75]. Likewise, the increased risk of cerebrovascular events in HIV-infected individuals [76, 77] is exaggerated in women [78]. Of note, the epidemiology of these comorbid conditions varies significantly across different social and geographic contexts obligating thoughtful design of trials to assess for the contribution of sex [79]. The differences in risk profile between men and women highlight the potential for studies of sex differences to identify causal pathways or biomarkers of disease pathogenesis.

## HIV Eradication and Functional Cure

The goal of HIV eradication or functional cure has become a focal point for HIV research. It is not known whether sex differences in viral and inflammatory set points in untreated infection translate into differences in ART-treated disease that have implications for HIV cure efforts. As women bear half the burden of the HIV epidemic, any intervention that will have a meaningful impact will need to be effective for both men and women. Importantly, several of the interventions in development for HIV cure are immunomodulatory [80]; this is an important divergence from the direct antiviral agents used in suppressive ART. Subtle immunologic differences between men and women may play a critical role in determining the safety and efficacy of curative interventions.

There are limited data defining sex differences in viral reservoir size and dynamics. Two cross-sectional studies with approximately 30% enrollment of women reported lower levels of HIV DNA in women [81, 82]. However, data from a prospectively enrolled cohort of ART-treated men and women did not show any significant difference in HIV DNA levels, but rather showed lower levels of residual viremia by single copy assay and lower levels of multiply-spliced cell associated HIV RNA from women (Scully et al., Abstract 281, CROI 2017). In general, conclusions are limited by the underrepresentation of women in studies relevant to cure [83]. Specifically, in seminal work comparing different methods of reservoir quantitation, there were no XX participants and only 2 of 30 are identified as transgender (male to female) without data about exogenous hormone exposure [84]. In studies assessing the role of HIV DNA in predicting viral rebound, cohorts have been 82–100% male [85–87]. Of participants in trials of the histone deacetylase (HDAC) inhibitor class of latency reversal agents, only 2 of 50 participants were women [88–91]. As mentioned above, curative interventions such as TLR agonists and exhaustion reversal with immune checkpoint inhibitors are primarily targeting host and not viral factors. Both the TLR7 agonist pathway [47] and the immune checkpoint inhibitor pathways [92, 93] have shown sex-specificity in other contexts that should be considered carefully in the development of clinical trials.

## Potential Mechanisms

**Fig. 1:**
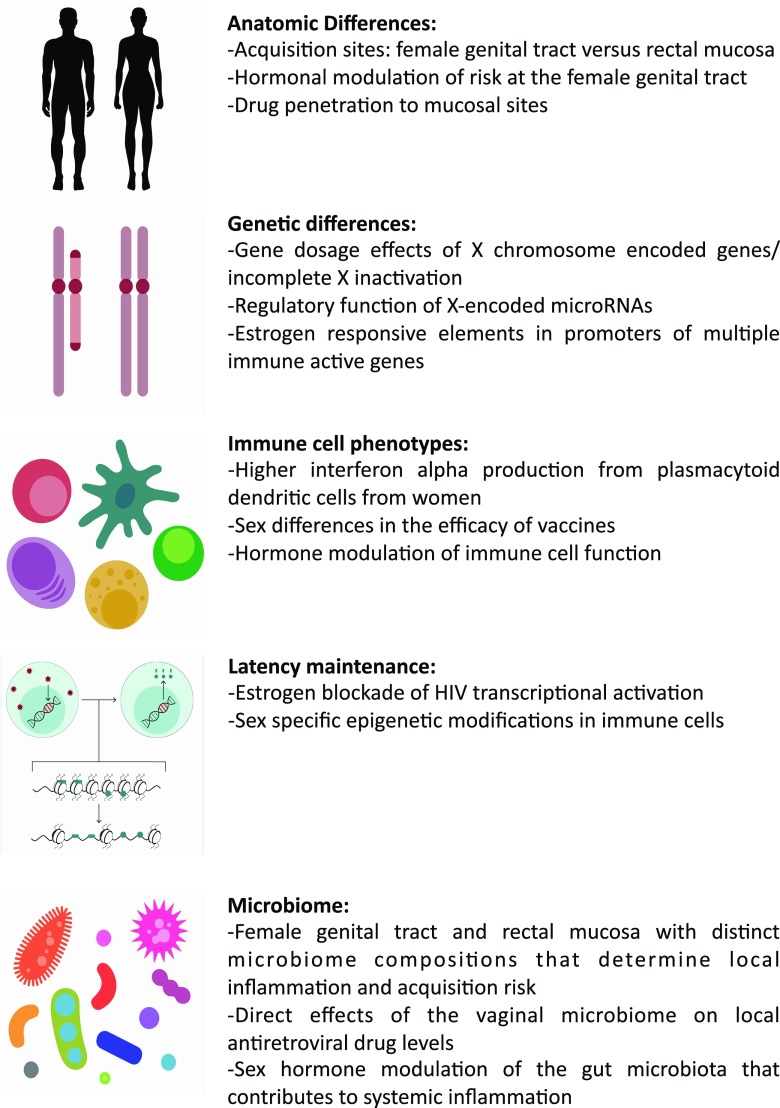
Summary of five critical domains of sex differences with relevance for HIV infection and potential or demonstrated mechanisms for their effects

Outlined above are multiple features of HIV acquisition, prevention, pathogenesis, and persistence that show sex variation. Behavioral and social characteristics differ between men and women, and these factors play an important role in sexual agency, reproductive health, and access to education and medical care. Indeed, sex-specific behaviors around adherence to interventions proved to be critical modifiers of the efficacy of PrEP [94]. Aside from these factors, there are a few domains of biological sex-specificity that are likely contributing to differences and can be exploited to therapeutic benefit (Fig. 1).

### Sex Hormone Effects

As noted above, there is an association with DMPA contraceptive use and enhanced rates of infection. The precise mechanisms are unclear, as the progestin-associated thinning of the vaginal mucosal observed in non-human primate models [95–97] has not been seen in women [98–102]. Recent data identified endogenous and exogenously administered progesterone-induced variations in the frequency of cervical HIV-susceptible target cells [103]. There are additional intersections between sex hormone levels and inflammation induced by microbiome composition and concurrent infections [104]. Given the global need for effective family planning methods and widespread use of hormonal contraception, determining the mechanisms of hormonal contribution to risk of infection and potential pathways for modification is of critical importance.

Outside of acquisition, estrogen is also a direct modifier of HIV transcription. Previous work has demonstrated that the estrogen receptor can be indirectly recruited to the HIV-1 long terminal repeat (LTR) and act to repress transcriptional activity [105]. More recently, using an unbiased small hairpin RNA screening strategy, the estrogen receptor was identified as a potent inhibitor of HIV transcription in latency models and primary cells (Karn et al., IAS, 2015; Das et al., submitted). Ex vivo studies using primary cells from both men and women confirmed that estrogen is repressive to latency reversal, and that blockade of the estrogen receptor can enhance reactivation (Karn et al., IAS 2015; Das et al., submitted).

Sex hormones have also been reported to have a variety of direct effects on immune cell function. Both estrogen and progesterone have been reported to modulate plasmacytoid dendritic cell IFNα secretion [47, 106–109]. Cytotoxic T cells from women have higher expression of inflammatory/cytotoxic pathways after ex vivo restimulation, and multiple genes in these pathways have estrogen responsive elements in their promoters [22]. Of note, the presence of estrogenic compounds in standard cell culture media components [110, 111] and the difficulty in replicating the in vivo balance of hormones with in vitro studies obligates careful interpretation of these studies. However, hormonal pathways can be safely modulated in vivo and offer a potential adjunctive therapeutic pathway that may be of use in studies of HIV cure.

### Microbiome

Sex-specificity of the microbiome composition in the genital tracts is one determinant of the local immune environment. Further, recent work identified novel features of this relationship, with specific microbiome components associated with alterations in wound healing [112] and direct microbial metabolism of tenofovir associated with reduced efficacy of PrEP in the female genital tract [33]. Aside from this direct role, animal studies have demonstrated that sex hormones impact microbiome composition in the gut, with implications for sex-specific susceptibility to autoimmunity [113, 114]. Studies have confirmed sex variation in gut microbial contents in humans [115–117] and further work will be necessary to determine if these differences have consequences for inflammation in HIV disease. Interventions to reshape the microbiome (e.g., with probiotics) may offer therapeutic benefits.

### Genetic Differences

The sex-specific chromosomal complement is an additional pathway to biological differences. The X chromosome carries critical immune genes including *TLR7*, which encodes a pathogen sensor, *FOXP3*, a transcription factor critical for regulatory immune responses, and 10% of all microRNAs which have pleiotropic regulatory roles [118].

As some sex differences including lower viral loads in females are present prior to the onset of puberty, non-hormonal mechanisms including genetics are likely to play a role [119]. Gene dosage effects are attenuated by X chromosome inactivation, but the enhanced risk of female predominant diseases such as systemic lupus erythematosus in phenotypic males with XXY karyotype suggests that this is incomplete [120]. Growing evidence demonstrates that up to 20% of X chromosome genes escape inactivation [121]; this has clinical implications, with recent work suggesting that these genes may determine a portion of sex-specific susceptibility to cancer [122]. The role of sex chromosome-encoded genes in differential vaccine responses, HIV pathogenesis, and cure efforts is undefined; it is notable that the HIV controllers genome-wide association study to assess for genetic determinants of spontaneous control was restricted to autosomes [123]. Studies to identify polymorphisms in sex chromosomal genes should be pursued.

Of note, research has also demonstrated sex-specific transcriptional programs related to both chromosomal determinants and ongoing hormonal programming [124]. Analysis of the methylation patterns and transcriptome of immune cell subsets identifies differences between men and women, supporting a potential role for epigenetic regulation in sex differences in immune responses [125]. Given the potential use of epigenetic modifiers in latency reversal, sex-specific patterns of epigenetic regulation should be explored.

### Immunological Differences

The combined effects of sex hormones, microbiome, and chromosomal complement contribute to distinct immune profiles. Preliminary work suggests that the relationship between residual virus activity and T cell activation and exhaustion phenotypes may be different between men and women, with men showing more activation and exhaustion and more correlations with measures of viral reservoir (Scully et al., Abstract 281,CROI 2017). Previous work has also demonstrated sex differences, partially mediated by estrogen, in antibody features including subclass, levels of hypermutation, and Fc glycan modifications [23, 24]. Sex-stratified comparisons of the humoral responses to vaccines may provide insight into the critical features of a successful response.

Also notable is the role of sex hormones in lipid metabolism that is in turn linked to innate cellular activation and risk of non-AIDS morbidity and mortality in HIV infection [126, 127]. Of note, recent data suggests that there may be sex-specific responses to lipid-lowering therapy, with women showing qualitatively greater reductions in sCD163 after treatment with pitavastatin [128]. In studies of soluble markers of inflammation, sex differences in baseline levels and in the changes after ART initiation have been reported; neopterin (marker of cellular activation associated with HIV-related neurocognitive disease) was higher in women with impaired cognition, a finding not observed in men alone, and TNF-RII was similarly elevated in cognitively impaired women but not in men [129]. In a heterogenous cohort of men and women from multiple global sites, women were reported to have lower baseline levels of CRP, lipopolysaccharide, and soluble CD14 (sCD14) but less decrease in CRP and sCD14 and more increase in TNFα after ART [71]. In contrast, in a more homogenous cohort comparison, women had lower CRP than men did at baseline but again showed limited change after initiation of ART [70]. In total, the data are far from definitive and the multiple determinants of inflammatory outcomes including coinfections, microbiome differences, sex hormones, and immune setpoints will need to be carefully parsed to guide interventions. What is clear is that sex is a modifier of immune responses and may also dictate which biomarkers are predictive of risk for a particular population.

## Gaps in Knowledge and Opportunities

Historically, there has been limited enrollment of women in clinical trials of HIV therapy in the developed world, a problem that has extended to the field of cure research [62, 83, 130]. Given the multiple lines of evidence for sex-based differences in immune responses [131], HIV disease pathogenesis [132], and pharmacokinetics/pharmacodynamics [133], it is imperative that biological sex is considered in the development and implementation of new clinical interventions; successful innovations will need to have efficacy in both men and women. Further, as discussed above, sex differences offer a comparator point that may elucidate pathways critical for robust immune responses or curative strategies that can be leveraged to therapeutic success in both sexes.

Although not the focus of this review, the intersection between genetic complement and sex hormone exposure is particularly highlighted in transgender individuals. Given the burden of HIV in transgender individuals [134] and growing evidence for the feasibility of high-quality studies in this population [135], HIV cure research needs to include transgender participants. Thoughtful comparative analysis may point to mechanistic links between the genetic complement and hormonal exposure and virologic and immunologic outcomes and will be critical to verify the safety and efficacy of proposed interventions.

Given the importance of analyzing the role of sex [136], what are the barriers to implementation? From the perspective of the investigator, the cyclic variation in hormone levels and/or exogenous hormone administration and potential for pregnancy introduce variables and safety concerns that can require larger sample sizes and more intensive monitoring of interventions. These concerns notwithstanding, the global burden of HIV infection in women and the population of women and girls at risk obligates that research specifically address the optimal treatment, prevention, and curative interventions for women [137]. From the view of the potential study participants, engagement with research, education about risks and benefits, and addressing logistical challenges to enrollment are all feasible [138]. Prior work has established that women can be successfully recruited and retained in HIV research [139, 140], and these experiences should be used to guide recruitment efforts. In addition, exploratory basic and clinical studies should report data by sex; while not always sufficient for a powered analysis, this data can be helpful in aggregate to determine when sex differences bear more focused investigation.

## Conclusion

Sex differences in HIV arise from the combinatorial effects of sex hormones, genetic differences, and sociobehavioral and environmental influences. These differences are clinically relevant, translating into enhanced risk for acquisition and non-AIDS morbidity in women, but also potentially for more efficacious immune responses to vaccination. The role of sex differences in cure interventions remains to be defined. Robust sex comparisons must be carefully controlled as enrollment of women tends to be preferentially in resource-limited settings introducing potentially confounding genetic and environmental differences when compared to predominantly male cohorts from the developed world. Despite these challenges, focused investigation of sex differences has uncovered important features of disease, highlighting pathogenic inflammatory pathways. The direct role of sex hormones in modulating immune subset distribution and HIV transcription exemplifies how this research can lead to therapeutic interventions with hormone receptor antagonists or specific selection of contraceptive preparations. Likewise, highlighting the immune pathways that differ between men and women may indicate mechanisms to optimize treatment responses with adjuvant or immunomodulatory interventions that target these pathways in the “weaker” sex, whichever that may be.
